# Comparative effectiveness and safety of sarilumab vs JAK inhibitors in late- and younger-onset rheumatoid arthritis

**DOI:** 10.1093/rheumatology/keag289

**Published:** 2026-06-08

**Authors:** Yuji Nozaki, Kazuya Kishimoto, Tetsu Itami, Daisuke Tomita, Yumiko Wada, Takuya Kotani, Tohru Takeuchi, Toshihiko Hidaka, Shoichi Hino, Toshiaki Miyamoto, Hirofumi Miyake, Kazunari Hatta, Kenji Mamoto, Yutaro Yamada, Tadashi Okano, Takaichi Okano, Jun Saegusa, Masahiro Horita, Keiichiro Nishida, Koji Kinoshita, Shinya Rai

**Affiliations:** Department of Hematology and Rheumatology, Kindai University Hospital, Osaka, Japan; Department of Hematology and Rheumatology, Kindai University Hospital, Osaka, Japan; Department of Hematology and Rheumatology, Kindai University Hospital, Osaka, Japan; Department of Hematology and Rheumatology, Kindai University Hospital, Osaka, Japan; Department of Internal Medicine (IV), Division of Rheumatology, Osaka Medical and Pharmaceutical University, Takatsuki, Japan; Department of Internal Medicine (IV), Division of Rheumatology, Osaka Medical and Pharmaceutical University, Takatsuki, Japan; Department of Internal Medicine (IV), Division of Rheumatology, Osaka Medical and Pharmaceutical University, Takatsuki, Japan; Rheumatology Center, Miyazaki Zenjinkai Hospital, Miyazaki, Japan; Department of Rheumatology and Clinical Immunology, Izumi City General Medical Center, Osaka, Japan; Miyamoto Clinic, Shizuoka, Japan; Department of Rheumatology and Clinical Immunology, Seirei Hamamatsu General Hospital, Hamamatsu, Japan; Department of General Internal Medicine, Tenri Hospital, Nara, Japan; Department of General Internal Medicine, Tenri Hospital, Nara, Japan; Department of Orthopaedic Surgery, Osaka Metropolitan University Graduate School of Medicine, Osaka, Japan; Center for Senile Degenerative Disorders (CSDD), Osaka Metropolitan University Graduate School of Medicine, Osaka, Japan; Center for Senile Degenerative Disorders (CSDD), Osaka Metropolitan University Graduate School of Medicine, Osaka, Japan; Department of Rheumatology and Clinical Immunology, Kobe University Graduate School of Medicine, Kobe, Japan; Department of Rheumatology and Clinical Immunology, Kobe University Graduate School of Medicine, Kobe, Japan; Department of Orthopaedic Surgery, Faculty of Medical Development Field, Okayama University, Okayama, Japan; Locomotive Pain Center, Faculty of Medical Development Field, Okayama University, Okayama, Japan; Department of Hematology and Rheumatology, Kindai University Hospital, Osaka, Japan; Department of Hematology and Rheumatology, Kindai University Hospital, Osaka, Japan; Centre for Lymphoid Cancer, British Columbia Cancer, Vancouver, BC, Canada

**Keywords:** RA, sarilumab, Janus kinase inhibitors, late-onset RA, younger-onset RA, drug retention, MTX, glucocorticoids

## Abstract

**Objectives:**

Glucocorticoid (GC)- and MTX-sparing strategies are clinically important in RA, yet comparative real-world evidence for sarilumab (SAR) vs Janus kinase inhibitors (JAKi) is limited. Whether treatment effects differ by age at onset—late-onset RA (LORA) vs young-onset RA (YORA)—also remains unclear. This study aimed to compare the effectiveness and safety of SAR and JAKi in patients with LORA and YORA.

**Methods:**

We conducted a multicentre real-world cohort study of adults with RA initiating SAR or a JAKi. Outcomes over 12 months included change from baseline in MTX dose (ΔMTX, mg/week) and GC dose (ΔGC, mg/day), cumulative treatment discontinuation and change in Clinical Disease Activity Index (CDAI). Analyses were performed within LORA and YORA strata, and propensity score matching (PSM) was applied separately in each stratum to balance baseline characteristics. Sensitivity analyses were conducted in the matched cohorts.

**Results:**

Both SAR and JAKi were associated with progressive tapering of concomitant MTX and GC during follow-up. SAR was associated with a higher frequency of MTX discontinuation compared with JAKi. In contrast, GC discontinuation and CDAI improvement were broadly similar between treatments across age strata and MTX subgroups. Findings were consistent after PSM and in sensitivity analyses within both LORA and YORA.

**Conclusion:**

In routine clinical practice, SAR and JAKi support de-escalation of concomitant therapy. SAR may permit earlier MTX withdrawal without loss of disease control, with comparable GC tapering and disease activity improvement in both LORA and YORA.

Rheumatology key messages

- Sarilumab showed comparable effectiveness to JAK inhibitors in patients with RA.
- Sarilumab had a favourable safety profile in late-onset RA.
- Sarilumab may be a useful treatment option for older patients with RA.

## Introduction

The prevalence of late-onset RA (LORA) is increasing, and optimizing treatment strategies in this population has become a pressing clinical need [1]. In routine practice, the ability to achieve and maintain remission or low disease activity (LDA) while de-escalating—ideally discontinuing—concomitant MTX and glucocorticoids (GCs) is central to improving both outcomes and safety [2, 3].

LORA poses distinctive challenges to de-escalation. Renal impairment is relatively common, often limiting MTX to moderate doses or precluding its use altogether [4]. In parallel, GC exposure elevates the risk of infections, and once initiated, GC can be difficult to withdraw in real-world care [5]. These constraints complicate the balance between disease control and medication minimization.

Among targeted therapies, IL-6 receptor inhibitors (IL-6Ri) and Janus kinase inhibitors (JAKi) are key options with rapid anti-inflammatory effects and potential GC-sparing benefits [6]. However, important evidence gaps remain regarding MTX/GC discontinuation. IL-6Ri, such as sarilumab (SAR), have demonstrated clinical efficacy both in combination with conventional synthetic DMARDs (csDMARDs) and monotherapy [7, 8]. For tocilizumab (TCZ), prospective trials have suggested that MTX withdrawal is feasible, yet replication in real-world settings is limited. For JAKi, real-world data specifically focusing on attainment of MTX and GC discontinuation are sparse, and direct evaluations restricted to LORA are rarer still.

To address these gaps, we conducted a real-world study with a primary focus on LORA that directly contrasts SAR (IL-6Ri) with JAKi within the same multicentre cohort, while also providing complementary analyses in younger-onset RA (YORA). We evaluated two pragmatic, patient-centered de-escalation endpoints at 12 months—MTX and GC discontinuation—as well as safety, quantified as incidence rates per 100 patient-years for overall and serious adverse events (AEs), serious infection and herpes zoster.

Our objective was to provide comparative, clinically actionable evidence for therapy selection in LORA by integrating effectiveness (MTX and GC de-escalation) with safety in everyday practice.

## Patients and methods

### Patients

We performed a multicentre observational study across 13 rheumatology centres in Japan, including university hospitals and specialized clinics. RA was diagnosed according to the 1987 American College of Rheumatology or the 2010 ACR/EULAR classification criteria [9, 10]. We selected SAR as the IL-6Ri comparator because the present study was designed as a contemporaneous real-world comparison with JAKi in the treatment era after SAR approval in Japan. In addition, unlike SAR, tocilizumab has greater treatment heterogeneity in routine practice, including both intravenous and subcutaneous formulations, with the subcutaneous regimen administered either every other week or weekly in Japan according to disease activity. To avoid additional complexity related to route and frequency of administration and to simplify interpretation of the comparative data, we restricted the IL-6Ri comparator to SAR. Patients were stratified according to age at disease onset into two groups: LORA (≥65 years) and YORA (<65 years). This classification was applied throughout all comparative analyses including treatment persistence, disease activity and safety outcomes.

### Study outcomes

Study outcomes were assessed over 12 months after initiation of SAR or JAKi. The primary outcome was 12-month drug retention, defined as continuation of the index SAR or JAKi without permanent discontinuation for any reason. Secondary outcomes included: (1) changes in Clinical Disease Activity Index (CDAI) from baseline at 3, 6 and 12 months, (2) proportions of patients achieving CDAI LDA (CDAI ≤10.0) and remission (CDAI ≤2.8) at these time points, (3) changes in daily oral GC dose and cumulative GC discontinuation and (4) safety outcomes, including overall AEs, serious AEs, serious infections and herpes zoster. Exploratory analyses comprised age-stratified comparisons between LORA and YORA, subgroup analyses according to concomitant MTX use and prior biologic or targeted synthetic DMARD exposure, and evaluations of baseline predictors of treatment discontinuation and MTX/GC de-escalation.

### Ethics approval

The study was conducted in accordance with the Declaration of Helsinki and was approved by the ethics committee of Kindai University School of Medicine (Approval No. 31–020), with approval also obtained at participating institutions. Written informed consent was waived at Kindai University through an opt-out process and was obtained at the other institutions.

### Statistical analysis

Propensity score matching (PSM) (1:1 nearest-neighbor, without replacement; calliper 0.2 S.D. of the logit) was used to balance baseline characteristics between the groups. The model included age, sex, disease duration, baseline CDAI, number of prior b/tsDMARDs and concomitant GC use. Balance was assessed using standardized mean differences (SMDs), with SMD <0.10 considered acceptable. Patients with missing matching variables were excluded. Treatment retention was analysed using Kaplan–Meier curves, log-rank tests and Cox proportional hazards models. GC dose changes were assessed with the Wilcoxon signed-rank test, and discontinuation rates with *χ*^2^ tests. Within matched cohorts, multivariable regression was used to adjust for residual imbalance. CDAI and GC changes were analysed on an observed-case basis without imputation after treatment discontinuation.

Twelve-month ΔCDAI was therefore calculated among patients with both baseline and 12-month CDAI available. For GC outcomes, patients receiving no GC at baseline were assigned a daily dose of 0 mg/day and were considered already off GC when calculating GC discontinuation proportions; changes in GC dose are presented as medians with interquartile ranges owing to skewed distributions. Incidence rates for safety outcomes were calculated as events per 100 patient-years with 95% CIs, assuming a Poisson distribution. No formal statistical testing of between-group differences in incidence rates was performed, and comparisons of incidence rates are interpreted descriptively. Safety outcomes were analysed in the overall age-stratified cohorts rather than in the propensity score-matched cohorts, because matching substantially reduced the sample size and event counts for descriptive safety analyses. Prespecified stratified analyses were performed by age group, concomitant MTX use and prior b/tsDMARD exposure; these analyses were exploratory, and no formal adjustment for multiple testing was applied. Sample size was determined by the number of eligible patients in this multicentre cohort, with no formal a priori calculation. All analyses were performed using JMP Pro 18.0 (SAS Institute, Cary, NC) and GraphPad Prism 10 (GraphPad Software, San Diego, CA).

## Results

### Patient characteristics

As summarized in Table 1, among LORA patients, 176 received SAR and 147 received JAKi before PSM, yielding 85 vs 85 patients after matching. Before matching, the JAKi group showed a lower inflammatory burden and more prior exposure to advanced therapies: median CRP was 0.9 mg/dl [IQR 0.1–3.3] vs 3.0 [1.0–6.5], ESR 39.5 mm/h [18.0–61.3] vs 65.0 [33.0–92.0] and disease duration 46.0 [16.0–93.0] vs 16.0 [5.0–60.5] months in JAKi vs SAR, respectively. Prior IL-6Ri (17.0 vs 9.7%) and prior JAKi (17.8 vs 6.3%) were more frequent, and full-dose use was less frequent (63.3 vs 98.2%) in the JAKi group. After matching, baseline characteristics were largely comparable, including age, joint counts, global assessments, CDAI (19.2 ± 8.4 vs 17.8 ± 9.9), HAQ-DI and most laboratory variables.

**Table 1:** Baseline characteristics of patients with late-onset and younger-onset RA treated with sarilumab or JAK inhibitors before and after propensity score matching.

	Before propensity matching			After propensity matching			Before propensity matching			After propensity matching		
	SAR: *n* = 176	JAKi: *n* = 147	SMD before matching	SAR: *n* = 85	JAKi: *n* = 85	SMD after matching	SAR: *n* = 247	JAKi: *n* = 451	SMD before matching	SAR: *n* = 247	JAKi: *n* = 247	SMD after matching
Age at onset	LORA			LORA			YORA			YORA		
Age at onset, years	73.5 [69.0–79.0]	73.0 [69.0–76.0]	0.078	72.0 [68.5–79.0]	74.0 [69.0–77.0]	0.28	50.0 [40.0–58.0]	48.0 [36.0–55.3]	0.14	49.0 [39.5–57.0]	48.0 [39.0–55.0]	0.08
Female (%)	75.6	72.8	0.06	74.1	75.3	0.02	81.0	82.6	0.04	84.2	82.4	0.04
Age, years	77.7 ± 5.8	77.6 ± 5.2	0.01	77.4 ± 6.0	78.0 ± 5.3	0.1	61.5 ± 13.1	57.9 ± 13.9	0.26	60.0 ± 12.6	60.0 ± 12.7	0.00
Disease duration, months	16.0 [5.0–60.5]	46.0 [16.0–93.0]	0.6	20.4 [6.5–82.5]	40.0 [11.5–80.5]	0.36	155.0 [52.6–265.0]	125.0 [63.0–213.0]	0.22	142.0 [40.0–238.0]	143.0 [71.0–222.5]	0.00
Naive (%)	64.2	34.9	0.6	65.9	32.9	0.69	34.8	19.0	0.36	32.1	14.6	0.42
Full dose (%)	98.2	63.3	0.98	100	67.1	0.99	99.5	74.0	0.81	100.0	74.6	0.82
Prior IL-6R inhibitor use (%)	9.7	17.0	0.21	1.2	20.0	0.64	13.8	20.2	0.17	13.3	21.8	0.22
Prior JAK inhibitor use (%)	6.3	17.8	0.35	10.6	15.5	0.14	9.7	18.2	0.24	6.7	21.8	0.44
RF (%), titer (IU/ml)	66.9, 41.0 [9.0–132.0]	75.2, 74.0 [11.0–359.3]	0.18/0.17	72.5, 40.0 [10.0–124.5]	71.8, 83.0 [10.0–406.5]	0.01	79.2, 67.0 [19.0–197.8]	78.8, 43.9 [12.0–205.8]	0.01/0.16	79.7, 67.0 [19.8–179.1]	81.6, 41.0 [14.0–274.0]	0.04
ACPA (%), titer (IU/ml)	59.8, 7.0 [0.0–193.9]	69.7, 54.6 [0.3–325.5]	0.20/0.24	59.3, 8.6 [0.0–180.8]	65.5, 44.7 [0.0–282.0]	0.12	82.7, 58.4 [10.0–285.4]	75.7, 56.3 [5.9–205.1]	0.17/0.01	84.3, 62.0 [12.5–262.0]	79.4, 57.7 [14.5–209.2]	0.02
CRP, mg/dl [IQR]	3.0 [1.0–6.5]	0.9 [0.1–3.3]	0.64	2.6 [0.7–6.1]	0.9 [0.1–2.4]	0.13	0.9 [0.2–2.8]	0.2 [0.0–1.4]	0.45	0.8 [0.2–2.6]	0.3 [0.0–1.8]	0.14
ESR, mm/h [IQR]	65.0 [33.0–92.0]	39.5 [18.0–61.3]	0.66	59.5 [32.5–91.3]	41.0 [18.0–62.0]	0.28	38.0 [19.0–67.8]	23.5 [10.0–48.3]	0.44	37.0 [17.0–67.5]	26.5 [12.0–55.0]	0.12
Tender joints, range 0–28 [IQR]	3.0 [1.0–6.8]	3.0 [1.0–5.0]	0.0	2.0 [1.0–5.0]	3.0 [1.0–5.0]	0.20	4.0 [1.3–8.0]	2.0 [1.0–7.0]	0.42	4.0 [2.0–7.0]	2.0 [1.0–7.0]	0.24
Swollen joints, range 0–28 [IQR]	4.0 [2.0–7.0]	4.0 [1.0–6.0]	0.0	3.0 [1.0–6.0]	4.0 [2.0–6.0]	0.27	4.0 [2.0–7.0]	4.0 [1.0–6.0]	0.0	3.0 [1.0–6.0]	3.0 [1.0–6.0]	0.33
Patient visual analogue scale, 0–100 mm	59.5 [38.3–79.0]	54.0 [30.0–78.0]	0.16	51.0 [28.5–75.0]	58.0 [40.0–80.0]	0.21	52.0 [28.3–73.0]	57.0 [33.8–74.0]	0.15	52.0 [28.5–73.0]	60.0 [40.0–75.5]	0.26
Physician visual analogue scale, 0–100 mm	57.0 [30.0–80.0]	46.0 [30.0–70.0]	0.23	50.0 [25.5–70.0]	50.0 [30.0–75.0]	0.0	44.0 [25.0–62.0]	50.0 [26.0–70.0]	0.19	43.0 [25.5–61.0]	57.0 [32.0–71.5]	0.50
CDAI	21.0 ± 11.7	18.6 ± 8.9	0.22	17.8 ± 9.9	19.2 ± 8.4	0.15	20.0 ± 11.7	19.3 ± 11.7	0.06	19.1 ± 10.2	20.1 ± 12.0	0.09
HAQ-DI, range 0–3	1.0 [0.4–1.8]	1.1 [0.1–1.8]	0.08	0.8 [0.3–1.3]	1.4 [0.4–1.9]	0.24	0.9 [0.3–1.5]	0.8 [0.3–1.4]	0.11	0.7 [0.4–1.4]	1.0 [0.4–1.5]	0.11
WBC/μl	7921.6 ± 2483.3	7615.0 ± 2807.1	0.11	7174.1 ± 2072.9	7602.6 ± 2535.1	0.18	7432.4 ± 2906.9	6716.8 ± 2251.1	0.27	7206.3 ± 2576.6	6846.5 ± 2282.3	0.14
Neutrophils/μl	5677.4 ± 2323.3	5224.7 ± 2399.6	0.23	4983.2 ± 1862.9	5131.7 ± 2264.9	0.07	5189.6 ± 2759.9	4486.9 ± 2126.3	0.28	4965.8 ± 2485.1	4575.0 ± 2175.0	0.16
Hb, g/dl	11.1 ± 1.6	11.7 ± 1.7	0.36	11.1 ± 1.7	11.7 ± 1.7	0.35	12.0 ± 1.5	12.2 ± 1.5	0.13	12.1 ± 1.5	12.2 ± 1.7	0.06
Plt, ×10^4^/μl	31.3 ± 12.5	25.0 ± 7.8	0.60	29.1 ± 12.4	24.5 ± 7.7	0.44	27.0 ± 9.9	27.0 ± 8.9	0.0	26.7 ± 9.8	26.7 ± 9.0	0.0
AST, IU/l	20.0 [16.0–25.0]	21.0 [17.3–28.0]	0.13	20.0 [17.0–24.5]	21.0 [17.0–26.0]	0.16	20.0 [16.0–25.0]	21.0 [17.0–29.0]	0.12	20.0 [16.0–25.0]	21.0 [17.0–30.0]	0.12
ALT, IU/l	14.0 [10.0–21.0]	13.0 [10.8–20.0]	0.13	15.0 [10.0–21.0]	13.0 [11.0–19.3]	0.27	14.0 [10.8–22.0]	17.0 [11.0–24.8]	0.32	16.0 [11.0–25.8]	17.0 [11.0–25.8]	0.09
Cr, mg/dl	0.8 ± 0.5	0.8 ± 0.3	0.0	0.8 ± 0.3	0.9 ± 0.3	0.33	0.8 ± 0.5	0.7 ± 0.4	0.22	0.7 ± 0.2	0.7 ± 0.4	0.00
eGFR (ml/min/1.73 m^2^)	66.0 ± 24.0	62.4 ± 20.8	0.16	64.8 ± 23.6	60.6 ± 20.9	0.18	73.3 ± 25.2	79.7 ± 22.6	0.26	74.2 ± 22.5	77.5 ± 21.9	0.14
MTX use (%)	35.6	34.1	0.03	42.4	30.6	0.25	42.5	61.4	0.39	45.5	59.4	0.28
MTX dose among MTX users, mg/week	6.9 ± 3.2	7.2 ± 2.7	0.10	6.6 ± 3.6	7.1 ± 2.7	0.15	7.5 ± 3.2	8.3 ± 3.0	0.26	7.5 ± 3.2	7.9 ± 2.8	0.13
JAKi: TOF/BAR/PEF/UPA/FIL (%)		15.7/20.4/12.9/24.5/26.5			14.1/21.2/14.1/29.4/21.2	0.0/0.0/0.0/0.0		24.4/30.2/9.3/22.1/14.0			24.2/30.3/10.3/20/15.2	0.0/0.0/0.0/0.0
csDMARDs: SASP/IGU/BUC/TAC (%)	27.8/23.9/4.6/5.7	34.7/40.8/8.8/8.2	0.14/0.36/0.16/0.09	32.9/24.7/5.9/5.8	38.8/35.3/3.5/8.2	0.15/0.12/0.05/0.14	25.9/27.1/4.9/9.7	26.4/26.0/4.7/7.4	0.01/0.02/0.00/0.08	30.9/30.9/6.1/9.1	24.2/25.5/4.9/5.5	0.12/0.23/0.11/0.09
Glucocorticoid (%), mg/day [IQR]	37.0, 0.0 [0.0–5.0]	47.0, 0.0 [0.0–5.0]	0.2/0.0	33.3, 0.0 [0.0–2.9]	45.9, 0.0 [0.0–5.0]	0.62/0.0	47.0, 0.0 [0.0–5.0]	45.3, 0.0 [0.0–5.0]	0.03/0.0	43.0, 0.0 [0.0–5.0]	44.9, 0.0 [0.0–4.0]	0.03/0.0
Steinbrocker stage I/II/III/IV	56.6/23.9/13.8/5.7	49.6/23.1/15.7/11.6	0.04	48.2/32.5/15.7/3.6	55.1/24.6/17.4/2.9	0.02	25.2/24.8/23.9/26.1	31.8/18.7/19.2/30.4	0.04	23.7/26.3/27.6/22.4	31.0/16.2/23.2/30.0	0.06
Steinbrocker class 1/2/3/4	19.1/45.4/29.0/6.6	24.1/47.4/24.1/4.5	0.04	26.6/48.1/19.0/6.3	20.8/42.9/31.2/5.2	0.02	24.2/51.5/21.7/2.6	22.9/54.7/21.0/1.4	0.05	23.9/56.6/17.6/1.9	19.0/54.9/25.4/0.7	0.15

Values are median [25th–75th centiles] or mean (S.D.), unless otherwise indicated.

Full dose was defined as: SAR 200 mg every 2 weeks; JAKi: TOF 10 mg/day, BAR 4 mg/day, PEF 150 mg/day, UPA 15 mg/day and FIL 200 mg/day. Reduced dose was defined as: SAR 150 mg every 2 weeks; JAKi: TOF 5 mg/day, BAR 2 mg/day, PEF 100 mg/day, UPA 7.5 mg/day and FIL 100 mg/day.

Abbreviations: ALT, alanine aminotransferase; AST, aspartate aminotransferase; BAR, baricitinib; BUC, bucillamine; CDAI, clinical disease activity index; Cr, creatinine; csDMARDs, conventional synthetic DMARDs; eGFR, estimated glomerular filtration rate; IGU, iguratimod; FIL, filgotinib; HAQ-DI, health assessment questionnaire disability index; Hb, haemoglobin; IL-6R, IL-6 receptor; IQR, interquartile range; JAKi, JAK inhibitors; LORA, late-onset RA (≥65 years); PEF, peficitinib; Plt, platelet; SAR, sarilumab; SASP, Salazosulfapyridine; SMD, standardized mean difference; TAC, tacrolimus; TOF, tofacitinib; UPA, upadacitinib; WBC, white blood cell; neutrophils, neutrophil count; YORA, younger-onset RA (<65 years).

Among YORA patients, 247 received SAR and 451 received JAKi before PSM, yielding 247 vs 247 patients after matching. Before matching, the JAKi group was younger and had milder disease activity: median age was 48.0 years [IQR 36.0–55.3] vs 50.0 [40.0–58.0], disease duration 125.0 [63.0–213.0] vs 155.0 [52.6–265.0] months, CRP 0.2 [0.0–1.4] vs 0.9 [0.2–2.8] mg/dl and ESR 23.5 [10.0–48.3] vs 38.0 [19.0–67.8] mm/h in JAKi vs SAR, respectively. Concomitant MTX use was more frequent in the JAKi group than in the SAR group (61.4 vs 42.5%). Among MTX users, the weekly MTX dose was also higher in the JAKi group (8.3 ± 3.0 vs 7.5 ± 3.2 mg/week). After matching, baseline characteristics were well balanced, including CDAI (19.1 ± 10.2 vs 20.1 ± 12.0), joint counts, global assessments, HAQ-DI and renal indices. Distribution of individual JAKi is detailed in Table 1, and overall non-age-stratified baseline characteristics are shown in Supplementary Table S1.

### Clinical outcomes

**Figure 1:**
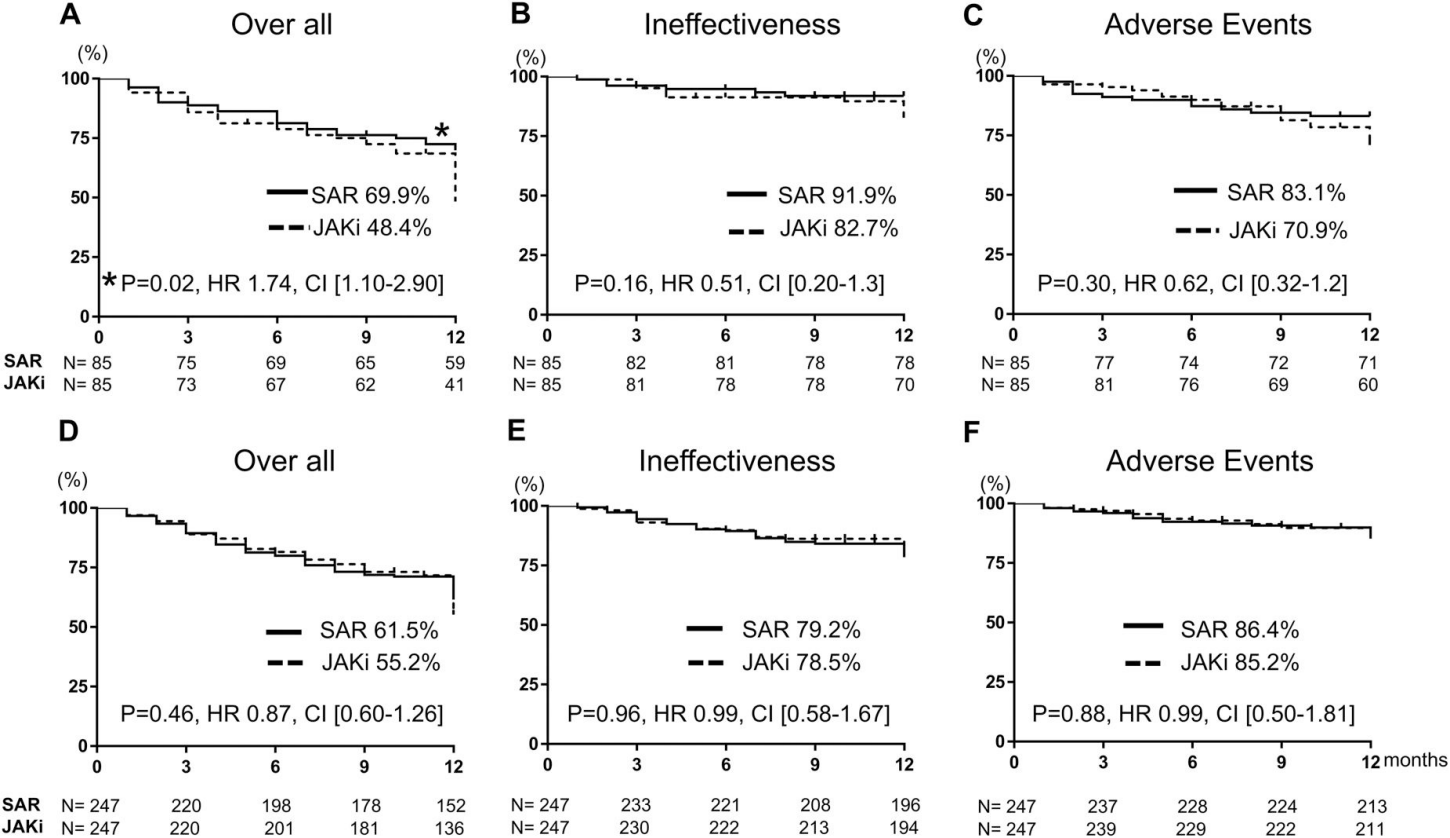
Comparative retention of SAR and JAKi across age strata (LORA vs YORA). Late-onset RA (LORA; ≥65 years) and younger-onset RA (YORA; <65 years) treated with SAR or JAKi. Panels (A–F) depict discontinuation outcomes for SAR or JAKi in LORA (≥65 years) and YORA (<65 years), with (A)–(C) corresponding to LORA—(A) all-cause discontinuation, (B) discontinuation due to ineffectiveness and (C) discontinuation due to adverse events—and (D)–(F) corresponding to YORA—(D) all-cause discontinuation, (E) discontinuation due to ineffectiveness and (F) discontinuation due to adverse events. Abbreviations: HR, hazard ratio; JAKi, Janus kinase inhibitors; LORA, late-onset RA (≥65 years); SAR, sarilumab; YORA, younger-onset RA (<65 years)

In LORA patients (Fig. 1A–C), Fig. 1A provides the groups’ overall retention rates, including all reasons for treatment discontinuation. At 12 months, the retention rates were 48.4% for the JAKi group and 69.9% for the SAR group. Kaplan–Meier survival analysis revealed the significant difference in treatment continuation between the groups (log-rank test, *P* = 0.02). Cox proportional hazards modelling demonstrated a statistically significant difference favouring SAR, with a hazard ratio (HR) of 1.74 (95% CI, 1.10–2.90). In the LORA subgroup, the wider separation of the overall retention curves near month 12 appeared to reflect clustering of discontinuation events in the JAKi group during the late follow-up period. Because overall discontinuation included reasons other than ineffectiveness or AEs, such as patient preference, transfer to another hospital or remission, this pattern should not be interpreted solely as reflecting differences in efficacy or safety. For ineffectiveness- and AEs-related discontinuation (Fig. 1B), SAR showed a numerical advantage without statistical significance: retention free from ineffectiveness was 91.9 vs 82.7% (*P* = 0.16, HR 0.51, 95% CI, 0.20–1.30) and was 83.1 vs 70.9% (Fig. 1C) (*P* = 0.30, HR 0.62, 95% CI, 0.32–1.20).

Among YORA patients (Fig. 1D–F), overall retention rates (Fig. 1D) did not differ (SAR 61.5% vs JAKi 55.2%, *P* = 0.46, HR 0.87, 95% CI, 0.60–1.26). ineffectiveness- and AEs-related discontinuation (Fig. 1E and F) was also similar (79.2 vs 78.5%; HR 0.99, 95% CI, 0.58–1.67 and 86.4 vs 85.2%; HR 0.99, 95% CI, 0.50–1.81).

### Longitudinal changes in CDAI according to onset age and MTX use with SAR and JAKi

**Figure 2:**
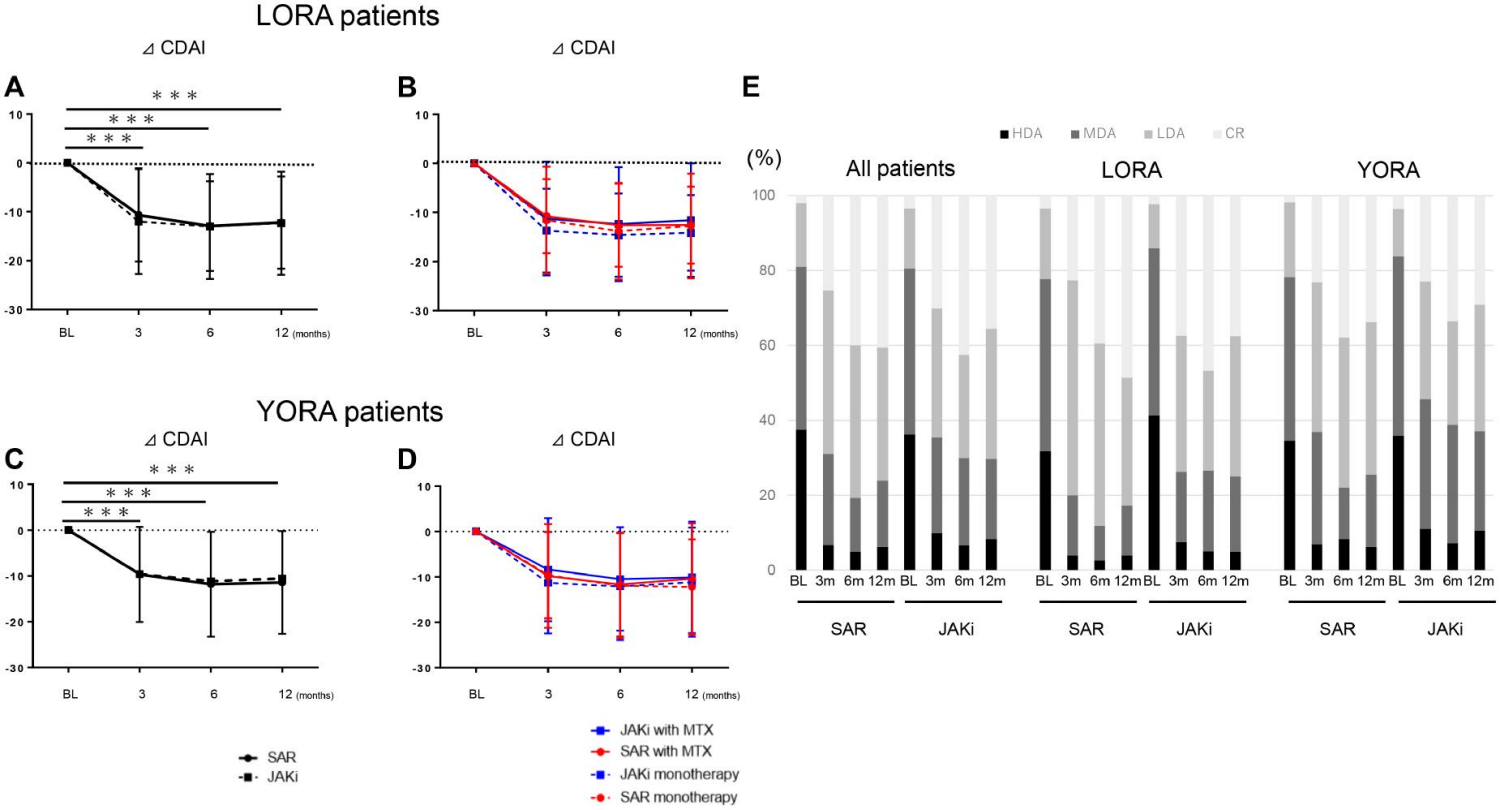
Longitudinal changes in disease activity according to age group and MTX use. Changes in CDAI over 12 months stratified by age category (LORA vs YORA) and treatment group (SAR vs JAKi). (A, B) CDAI improvement from baseline (ΔCDAI) in LORA patients: (A) overall, and (B) further stratified by MTX use (combination therapy vs monotherapy) within the SAR and JAKi groups. (C, D) Corresponding ΔCDAI analyses in YORA patients: (C) overall, and (D) further stratified by MTX use. In the MTX-stratified analyses, the numbers of patients receiving monotherapy were *n* = 36 and *n* = 53 in the SAR and JAKi groups, respectively, in LORA and *n* = 142 and *n* = 95 in YORA. (E) Distribution of CDAI disease activity categories (remission ≤2.8, low ≤10, moderate ≤22 and high >22) at baseline, 3 months, 6 months and 12 months for each treatment group. Both SAR and JAKi demonstrated significant CDAI improvement across age and MTX strata (***P < 0.001 vs baseline). Error bars indicate standard error of the mean. Abbreviations: CDAI, Clinical Disease Activity Index; JAKi, Janus kinase inhibitors; LDA, low disease activity; LORA, late-onset RA (≥65 years); SAR, sarilumab; YORA, younger-onset RA (<65 years)

Fig. 2 summarizes longitudinal changes in CDAI (ΔCDAI) according to age at onset, treatment group and concomitant MTX use over 12 months. In LORA patients (Fig. 2A), baseline CDAI was comparable between the two groups (SAR 17.8 ± 9.9 vs JAKi 19.2 ± 8.4, *P* = 0.07), and both the groups showed marked improvement through 12 months (ΔCDAI at 12 months: −12.3 ± 10.6 and −12.2 ± 9.5, respectively; both *P* < 0.001 vs baseline). Stratified analysis by MTX use in LORA (Fig. 2B) showed no significant difference in CDAI improvement between combination therapy and monotherapy in either the JAKi group (−14.1 ± 7.7 vs −11.6 ± 11.6 at 12 months, *P* = 0.56) or the SAR group (−13.3 ± 8.1 vs −11.4 ± 10.3, *P* = 0.05). Similar findings were observed in YORA patients (Fig. 2C and D). Baseline CDAI was also comparable between the groups (SAR 19.1 ± 10.2 vs JAKi 20.1 ± 12.0, *P* = 0.78), and the mean improvement at 12 months was similar (−10.5 ± 12.1 with SAR vs −11.4 ± 11.3 with JAKi, *P* = 0.19). Concomitant MTX use did not significantly affect ΔCDAI in either treatment group.

Fig. 2E shows the longitudinal distribution of CDAI disease activity categories. In both LORA and YORA, the proportions of patients achieving remission or low disease activity increased progressively over time, whereas the proportions remaining in moderate or high disease activity decreased substantially. At 12 months, remission rates were 37.5% with JAKi and 48.7% with SAR in LORA, and 29.1 and 33.8%, respectively, in YORA (*P* = 0.68). The corresponding rates of low disease activity were 37.5 vs 34.2% in LORA and 33.8 vs 40.7% in YORA (*P* = 0.57). No significant between-group differences were observed at either 6 or 12 months.

### Changes in MTX and GC dose and cumulative discontinuation: JAKi compared with SAR

**Figure 3:**
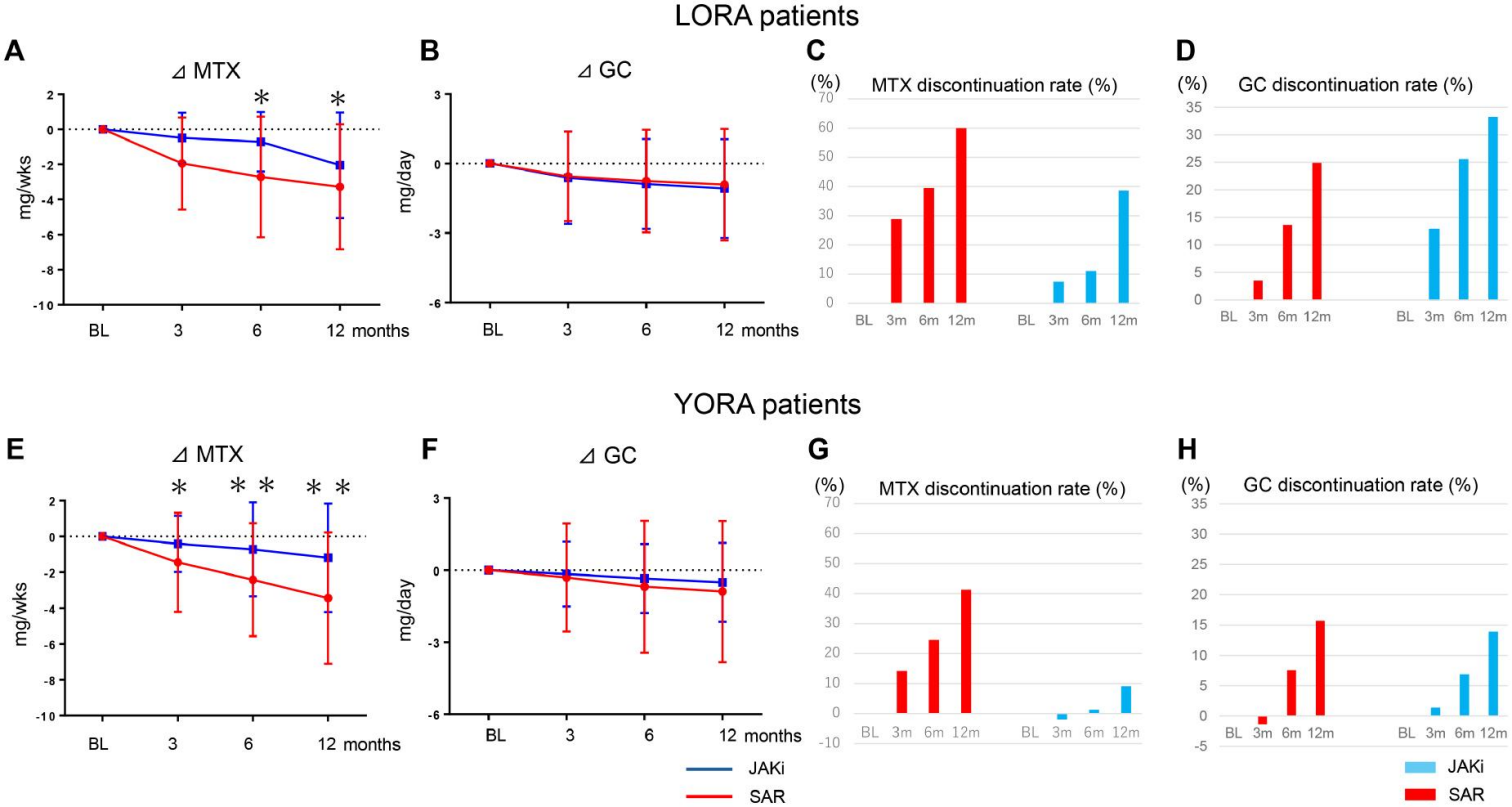
Changes in MTX and GC dose and cumulative discontinuation over 12 months stratified by age group (LORA vs YORA) and treatment (SAR vs JAKi). (A–D) LORA: (A) change from baseline in weekly MTX dose (ΔMTX, mg/week), (B) change from baseline in daily GC dose (ΔGC, mg/day), (C) cumulative MTX discontinuation (%) and (D) cumulative GC discontinuation (%) at baseline, 3 months, 6 months and 12 months for SAR and JAKi. (E–H) YORA: (E) ΔMTX, (F) ΔGC, (G) cumulative MTX discontinuation and (H) cumulative GC discontinuation at the same time points for SAR and JAKi. Asterisks indicate significant between-group differences between the two indicated time points. Error bars indicate standard error of the mean. MTX is expressed in mg/week and GC in mg/day. Small negative % near 0% arise from rounding and are treated as 0% in text and annotations. Abbreviations: BL, baseline; GC, glucocorticoid; JAKi, Janus kinase inhibitors; LORA, late-onset RA (≥65 years); SAR, sarilumab; YORA, younger-onset RA (<65 years)

Fig. 3 shows 12-month changes in MTX and GC dose and cumulative discontinuation in LORA and YORA patients treated with SAR or JAKi. In LORA, both the groups tapered MTX, with a greater reduction with SAR (−1.9, −2.7 and −3.2 mg/week at 3, 6 and 12 months) than with JAKi (−0.5, −0.7 and −2.0 mg/week), and between-group differences were significant at 6 and 12 months (*P* < 0.05) (A). GC doses declined in parallel without a material between-group difference (SAR −0.6, −0.9 and −1.1 mg/day; JAKi −0.6, −0.8 and −0.9 mg/day) (B). Cumulative MTX discontinuation increased and remained higher with SAR (0, 29.0, 39.5 and 60.1%) than with JAKi (0, 7.4, 11.1 and 38.6%) (C), while cumulative GC discontinuation rose in both the groups with broadly comparable levels (SAR 3.5, 13.6 and 24.9%; JAKi 12.8, 25.6 and 33.3%) (D). In LORA, baseline GC users in both the treatment groups showed substantial tapering, and >40% had completely discontinued GCs by 12 months.

In YORA, MTX declined in both the groups with a larger early and sustained reduction with SAR (−1.4, −2.4 and −3.4 mg/week) than with JAKi (−0.4, −0.7 and −1.1 mg/week), with significant differences at 3 months (*P* < 0.05) and at 6 and 12 months (*P* < 0.01) (E). GC doses decreased over time in both the groups without a meaningful between-group difference (SAR −0.3, −0.7 and −0.9 mg/day; JAKi −0.2, −0.3 and −0.5 mg/day) (F). Cumulative MTX discontinuation increased and remained higher with SAR (0, 14.3, 24.6 and 41.3%) than with JAKi (0, ∼0% due to rounding, 1.3 and 9.1%) (G). The change from baseline in cumulative GC discontinuation progressed similarly in both the groups (SAR −1.4, 7.6 and 15.7 percentage points; JAKi 1.4, 6.9 and 13.9 percentage points at 3, 6 and 12 months) (H).

### Safety outcomes and AEs lead to treatment discontinuation

Table 2 summarizes safety outcomes during treatment with SAR and JAKi. Rates of any AEs (17.8 vs 20.0 events per 100 patient-years) and serious AEs (8.9 vs 10.4) were comparable between SAR and JAKi. Serious infections were numerically less frequent with SAR (3.2 vs 4.6), whereas herpes zoster was markedly less common with SAR (0.5 vs 4.0). Malignancy and cardiovascular events were rare, and renal or hepatic injury was uncommon overall, although renal injury occurred more often with JAKi. Bone-marrow suppression appeared somewhat more frequent with SAR.

**Table 2:** Safety profile with SAR and JAK inhibitors treatment information of all RA patients.

	Sarilumab	JAK inhibitors
	Incident rate per 100 patient-yr (95% CI)	Incident rate per 100 patient-yr (95% CI)
All patients		
Any adverse events	17.8 (14.0–22.4)	20.0 (15.6–25.3)
Serious adverse events	8.9 (6.7–11.0)	10.4 (7.7–13.0)
Serious infection	3.2 (1.7–5.4)	4.6 (2.7–7.5)
Infection	5.4 (3.9–7.7)	5.2 (3.1–8.2)
Herpes zoster	0.5 (0.1–1.8)	4.0 (1.8–6.1)
Cancer	2.4 (1.2–4.5)	2.3 (1.0–4.6)
Cardiovascular event	0.0 (0.0–0.0)	0.3 (0.0–1.6)
Exacerbation of RA-ILD	1.5 (0.5–3.2)	1.2 (0.3–3.0)
Renal injury	0.0 (0.0–0.0)	2.5 (1.5–4.4)
Hepatic injury	0.5 (0.1–1.8)	1.2 (0.3–3.0)
Bone marrow suppression	3.9 (2.5–6.1)	1.3 (1.0–2.6)
LORA		
Any adverse events	23.2 (15.9–32.8)	29.6 (20.1–41.9)
Serious adverse events	10.5 (5.2–18.8)	13.1 (7.7–20.6)
Serious infection	3.6 (1.2–8.4)	5.7 (2.1–12.5)
Infection	7.1 (4.0–12.5)	6.8 (3.5–13.1)
Herpes zoster	0.7 (0.0–4.0)	4.9 (3.2–9.9)
Cancer	4.4 (1.6–9.5)	3.9 (1.6–9.4)
Cardiovascular event	0.0 (0.0–0.0)	0.5 (0.1–1.3)
Exacerbation of RA-ILD	1.5 (0.2–5.2)	1.8 (1.0–7.8)
Renal injury	0.0 (0.0–0.0)	4.8 (1.5–11.1)
Hepatic injury	1.1 (0.6–1.8)	1.2 (0.3–3.6)
Bone marrow suppression	4.3 (2.1–8.8)	3.0 (0.4–10.8)
YORA		
Any adverse events	14.0 (12.5–16.1)	16.9 (15.0–19.2)
Serious adverse events	7.3 (3.5–12.7)	7.7 (3.7–13.3)
Serious infection	2.2 (0.6–5.5)	5.7 (2.8–10.2)
Infection	4.1 (3.1–6.9)	4.2 (2.5–8.5)
Herpes zoster	0.0 (0.0–0.7)	2.5 (1.7–3.8)
Cancer	1.6 (0.3–4.7)	1.6 (0.3–4.5)
Cardiovascular event	0.0 (0.0–0.0)	0.0 (0.0–0.0)
Exacerbation of RA-ILD	1.5 (0.2–5.2)	1.8 (1.0–7.8)
Renal injury	0.0 (0.0–0.0)	0.0 (0.0–0.0)
Hepatic injury	0.0 (0.0–0.0)	1.6 (0.3–4.5)
Bone marrow suppression	3.8 (1.5–7.7)	0.0 (0.0–0.0)

Safety analyses were performed in the overall cohorts within each age stratum and were not restricted to the propensity score-matched population.

Abbreviations: AEs, adverse events; ILD, interstitial lung disease; JAKi, Janus kinase inhibitors; LORA, late-onset RA (≥65 years); SAR, sarilumab; YORA, younger-onset RA (<65 years).

In LORA patients, AE rates were higher overall but remained acceptable for both agents. Serious infections (3.6 vs 5.7) and herpes zoster (0.7 vs 4.9) were more frequent with JAKi, and renal events were observed only in the JAKi group. In YORA patients, overall AE rates were lower, and herpes zoster was not observed with SAR but occurred with JAKi (2.5). Overall, both SAR and JAKi showed acceptable safety profiles across age strata.

### Factors associated with discontinuation in SAR and JAKi within 12 months

In age-stratified multivariable logistic regression in Table 3, no baseline covariate reached statistical significance in the SAR cohort with LORA patients; notably, concomitant GC use showed no independent association with discontinuation (OR 1.24, 95% CI, 0.84–5.64; *P* = 0.21). By contrast, in the JAKi cohort with LORA patients, concomitant GC use was independently associated with higher odds of discontinuation (OR 2.71, 95% CI, 1.30–6.67; *P* = 0.03). Other covariates—including age, sex, disease duration, CDAI, RF/ACPA serostatus and concomitant MTX—were not significant in either treatment group within this stratum.

**Table 3:** Multivariate analysis was used to identify factors associated with discontinuation in SAR and JAKi within 12 months

	SAR		JAKi	
	**Multivariable analysis**		**Multivariable analysis**	
	**Odds ratio (95% CI)**	***P*-value**	**Odds ratio (95% CI)**	***P*-value**
LORA				
Age, years	0.97 (0.86–1.09)	0.64	0.98 (0.90–1.08)	0.38
Female	2.16 (0.46–10.1)	0.31	1.74 (0.62–4.85)	0.29
Disease duration, months	1.01 (0.99–1.03)	0.13	1.00 (0.99–1.02)	0.38
CDAI	0.99 (0.93–1.04)	0.60	0.68 (0.06–7.49)	0.74
RF positivity	1.07 (0.17–6.55)	0.94	2.85 (0.45–17.9)	0.26
ACPA positivity	1.91 (0.38–9.67)	0.43	0.26 (0.04–1.57)	0.14
Concomitant of GC	1.24 (0.84–5.64)	0.21	2.71 (1.30–6.67)	0.03
Concomitant of MTX	1.69 (0.47–6.08)	0.42	1.81 (0.72–4.51)	0.20
YORA				
Age, years	1.07 (0.98–1.03)	0.94	2.30 (0.99–1.04)	0.09
Female	0.62 (0.26–1.51)	0.29	0.89 (0.36–2.18)	0.79
Disease duration, months	1.07 (0.16–7.15)	0.94	1.17 (0.26–5.23)	0.82
CDAI	1.28 (0.28–5.91)	0.75	1.03 (1.00–1.06)	0.09
RF positivity	5.92 (1.48–13.6)	0.01	1.91 (0.60–6.10)	0.27
ACPA positivity	0.53 (0.18–1.55)	0.24	0.80 (0.25–2.56)	0.71
Concomitant of GC	1.81 (0.87–3.77)	0.10	0.75 (0.38–1.51)	0.10
Concomitant of MTX	0.56 (0.26–1.20)	0.14	0.67 (0.32–1.40)	0.28

Multivariable logistic regression analyses of factors associated with drug discontinuation within 12 months in patients treated with SAR or JAKi. Odds ratios and 95% CIs are presented.

Abbreviations: AEs, adverse events; CDAI, clinical disease activity index; GC, glucocorticoid; JAKi, Janus kinase inhibitors; LORA, late-onset RA (≥65 years); SAR, sarilumab; YORA, younger-onset RA (<65 years).

Among YORA patients, RF positivity was independently associated with higher discontinuation in the SAR cohort (OR 5.92, 95% CI, 1.48–13.6; *P* = 0.01). Concomitant GC use suggested a non-significant protective trend in JAKi, and MTX use was not associated with discontinuation due to AEs in either treatment group.

## Discussion

This multicentre, propensity score-matched cohort study provides real-world comparative evidence on the efficacy and safety of SAR and JAKi in patients with LORA. Previous studies have reported that JAKi exhibit superior GC-sparing effects compared with tumour necrosis factor inhibitors and are effective even in patients with poor prognostic factors, such as high ACPA titres and RF positivity [6]. Both agents demonstrated comparable improvements in disease activity and GC tapering. However, SAR showed significantly higher 12-month treatment retention in elderly patients and in those receiving early-line therapy, defined as first- or second-line b/tsDMARD treatment. In addition, overall treatment retention is a composite end point that may be influenced not only by ineffectiveness and AEs, but also by non-efficacy-related or non-safety-related reasons for discontinuation, including patient preference, transfer of care or remission. Therefore, the late separation of the overall retention curves in LORA should be interpreted cautiously. This difference was not evident in YORA, suggesting that age-related pharmacodynamic or safety factors may contribute to treatment continuity. Among LORA patients, treatment retention was significantly higher with SAR than with JAKi, indicating that IL-6Ri can provide sustained therapeutic benefit even in older populations. In contrast, discontinuations due to AEs were more frequent in the JAKi group, possibly related to higher concomitant GC use and prior exposure to IL-6Ri. In JAKi-treated LORA patients, prior exposure to IL-6R inhibitors was somewhat more frequent than in those starting SAR, reflecting real-world treatment sequences. However, the pattern of AE-related discontinuations did not point to a specific signal confined to patients with prior IL-6Ri use, and the limited number of events precludes firm conclusions about the contribution of treatment sequence to the higher AE-related discontinuation rates observed with JAKi. In JAKi-treated LORA patients, concomitant GC use at baseline was independently associated with a higher risk of treatment discontinuation. This likely reflects confounding by indication, because persistent GC requirement tends to mark patients with more refractory disease, a greater burden of comorbidities or increased frailty, all of which may lower the threshold for stopping JAKi when AEs or insufficient response occur. In addition, GC exposure itself may augment infectious risk, further contributing to discontinuation in this vulnerable population and underscoring the importance of GC-sparing strategies in elderly RA. Longitudinal CDAI trajectories and rates of remission or LDA were comparable between the groups, confirming that both IL-6 and JAK pathway blockade achieve similar disease control in clinical practice. The efficacy of SAR monotherapy has been demonstrated in phase 3 MONARCH trial, in which SAR monotherapy achieved significantly greater reductions in disease activity and higher ACR response rates than adalimumab monotherapy among MTX-intolerant patients [11]. Furthermore, the influence of MTX combination or tapering on IL-6 inhibition has been explored in studies with tocilizumab. In the Japanese SURPRISE trial, adding tocilizumab to MTX achieved earlier suppression of inflammation and radiographic progression than switching to monotherapy [12]. Conversely, in the ACT-TAPER trial, gradual MTX dose reduction in responders-maintained disease control at non-inferior rates compared with steady-dose MTX [13]. Collectively, these findings indicate that while IL-6R blockade benefits from MTX co-administration for rapid anti-inflammatory and structural protection, long-term disease control can be sustained even with MTX reduction or withdrawal. Therefore, both SAR and JAKi represent flexible treatment options applicable to patients’ intolerant to MTX or with renal impairment, as well as those suitable for MTX combination therapy. Because the biologic comparator was restricted to SAR, the present findings should be interpreted as specific to SAR and should not be generalized to all IL-6R inhibitors, including tocilizumab. In contrast to GC tapering, which is generally encouraged once disease control is achieved, MTX reduction in routine practice is less standardized and may reflect multiple individualized considerations, including intolerance, renal dysfunction, older age, comorbidities, patient preference and sustained disease control after initiation of the targeted agent. Therefore, the observed difference in MTX discontinuation between the groups should be interpreted as reflecting real-world treatment decisions rather than a protocol-driven tapering strategy.

GC discontinuation increased over time and did not differ between the treatment groups. Notably, >40% of LORA patients achieved complete GC withdrawal by 12 months, reflecting successful realization of the EULAR recommendation for early tapering. Given the well-recognized infection and metabolic risks of chronic GC exposure, these results underscore the clinical importance of GC reduction in elderly RA management.

In terms of safety, the overall and serious AE rates were comparable between SAR and JAKi; however, class-specific differences were observed. Herpes zoster occurred markedly more frequently with JAKi (4.9 events per 100 patient-years in LORA) compared with SAR (0.7 events per 100 patient-years). This observation aligns with prior global pooled analyses [14] and real-world Japanese registry data [15], both consistently showing that JAK inhibitors carry a higher risk of viral reactivation than biologic DMARDs targeting IL-6. Serious infections were slightly less frequent with SAR, whereas mild neutropenia occurred more often but was clinically manageable. Hepatic and renal AEs were rare, and cardiovascular event rates did not increase in either group. Overall, these findings suggest a favourable safety balance for SAR in elderly RA patients with elevated infection risk.

Multivariable analysis revealed that concomitant GC use was independently associated with treatment discontinuation in JAKi with LORA patients (OR 2.71, 95% CI, 1.30–6.67), whereas no significant predictors were identified in the SAR group. In younger patients, RF positivity was associated with SAR discontinuation (OR 5.92, 95% CI, 1.48–13.6), implying reduced responsiveness to IL-6 blockade in autoantibody-positive RA.

This study has several limitations. First, residual confounding cannot be excluded despite PSM and further multivariable adjustment. Second, longitudinal data on subsequent dose reduction or interval prolongation of SAR/JAKi were not systematically captured and may have influenced MTX de-escalation. Third, CDAI and GC trajectories were analysed on an observed-case basis without imputation after treatment discontinuation, so informative missingness is possible. Fourth, safety analyses were limited by small numbers of events and were not powered for definitive between-group comparisons. Fifth, stratified and subgroup analyses were prespecified but exploratory and were not adjusted for multiple comparisons. Finally, because the study was conducted in Japanese specialized centres, extrapolation to other populations or healthcare systems should be made with caution.

Taken together, IL-6Ri provided durable efficacy with lower infection risk, whereas JAKi remained an effective option, particularly in younger or refractory cases. These results emphasize that treatment selection should consider not only MTX tolerance but also age, infection susceptibility and GC exposure.

## Conclusion

In this real-world, multicentre study of LORA, SAR and JAKi demonstrated comparable efficacy in disease control and GC tapering irrespective of MTX use. However, SAR showed superior treatment persistence and a lower incidence of herpes zoster, suggesting a more favourable safety profile in elderly patients. These findings highlight the need for individualized therapeutic strategies based on age, infection risk and MTX tolerability.

## Supplementary Material

keag289_Supplementary_Data

## Data Availability

The datasets used and analysed in this study are available from the corresponding author upon reasonable request.
