# Supplementary material for: Comparative effectiveness and safety of sarilumab vs JAK inhibitors in late- and younger-onset rheumatoid arthritis
Source: Rheumatology (Oxford). 2026 Jun 8;65(6):keag289. doi: 10.1093/rheumatology/keag289 (PMC13275124; doi:10.1093/rheumatology/keag289)
Supplement: keag289_Supplementary_Data [file keag289_supplementary_data.docx]

**Supplementary Table S1.** Baseline characteristics of the overall rheumatoid arthritis cohort treated with sarilumab or JAK inhibitors

|  | **SAR: n=423** | **JAKi: n=598** | **p-value** |
| --- | --- | --- | --- |
| Age, years | 67.2 ± 13.7 | 64.7 ± 15.0*. | <0.05 |
| Female (%) | 78.2 | 81.0 | 0.27 |
| Disease duration, months | 89.0 [19.0–190.0] | 95.0 [38.0–189.3] | 0.05 |
| 1st/2nd/3rd/others (%) | 44.1/25.6/15.0/15.3 | 28.4/28.8/23.0/19.8*** | <0.001 |
| Full dose (%) | 98.9 | 69.6*** | <0.001 |
| Prior IL-6R inhibitor use (%) | 11.3 | 17.7** | <0.01 |
| Prior JAK inhibitor use (%) | 7.0 | 17.3 | <0.001 |
| RF (%), titer (IU/mL) | 74.5, 52.6 [14.5-172.0] | 76.6, 45.3 [11.8-222.8] | 0.52, 0.48 |
| ACPA (%), titer (IU/mL) | 75.7, 45.0 [1.0-277.5] | 74.0, 56.4 [2.6-269.1] | 0.62, 0.32 |
| CRP, mg/dL [IQR] | 1.4 [0.2–4.0] | 0.3 [0.1–1.9]*** | <0.001 |
| ESR, mm/hr [IQR] | 45.0 [22.0–76.0] | 27.0 [13.0–54.8]*** | <0.001 |
| Body mass index | 21.9 ± 3.5 | 22.6 ± 4.0 | 0.11 |
| Tender joints, range 0–28 [IQR] | 3.0 [1.0–7.0] | 3.0 [1.0–7.0] | 0.08 |
| Swollen joints, range 0–28 [IQR] | 4.0 [2.0–7.0] | 3.0 [1.0–6.0]** | <0.01 |
| Patient visual analogue scale, 0–100 mm | 52.0 [28.0–75.0] | 53.0 [30.0–74.0] | 0.95 |
| Physician visual analogue scale, 0–100 mm | 47.0 [28.0–67.0] | 44.5 [25.0–67.0] | 0.25 |
| CDAI | 20.7 ± 12.1 | 19.4 ± 11.1 | 0.26 |
| HAQ-DI, range 0–3 | 1.0 [0.4–1.7] | 0.6 [0.0–1.5]*** | <0.001 |
| WBC, /μL | 7659.2 ± 2770.1 | 7022.9 ± 2487.6** | <0.01 |
| Neutrophils, /μL | 5411.5 ± 2624.2 | 4737.4 ± 2246.7*** | <0.001 |
| Hb, g/dL | 11.6 ± 1.6 | 12.5 ± 0.6* | <0.05 |
| Plt, ×10⁴/μL | 28.8 ± 11.2 | 26.3 ± 8.6*** | <0.001 |
| AST, IU/L | 20.0 [16.0-24.5] | 21.0 [17.0-28.0]* | 0.06 |
| ALT, IU/L | 14.0 [10.0-21.0] | 15.0 [11.0-22.0] | 0.06 |
| Cr, mg/dL | 0.9 ± 0.4 | 0.8 ± 0.3 | 0.0 |
| eGFR (mL/min/1.73m^2^) | 70.4 ± 25.7 | 73.5 ± 23.4 | 0.16 |
| MTX use (%) | 41.9 | 53.5 | <0.001 |
| MTX dose among MTX users, mg/week | 7.9 ± 3.3 | 8.2 ± 3.0 | 0.31 |
| JAKi: TOF/BAR/PEF/UPA/FIL (%) |  | 22.7/30.6/10.9/19.9/16.0 |  |
| csDMARDs: SASP/IGU/BUC/TAC (%) | 23.7/31.7/4.6/8.5 | 31.7*/33.1/7.1/8.3 | <0.05 |
| Glucocorticoid (%), mg/day [IQR] | 40.9, 0.0 [0.0–5.0] | 45.3, 1.0 [0.0–4.0] | 0.24, 0.83 |
|  |  |  |  |
| Steinbrocker stage I/II/III/IV | 30.5/27.7/20.2/21.6 | 34.6/21.2/19.4/24.8 | 0.1 |
| Steinbrocker class 1/2/3/4 | 24.0/49.3/23.6/3.2 | 25.6/51.2/20.4/2.7 | 0.6 |

Values are median [25th–75th centiles] or mean (SD), unless otherwise indicated, SAR: Sarilumab, IL-6R: interleukin-6 receptor, JAKi: JAK inhibitors, RF: rheumatoid factor, ACPA: anticitrullinated peptide antibody, CRP: C-reactive protein, ESR: erythrocyte sedimentation rate, IQR: interquartile range**,** CDAI: clinical disease activity index, HAQ-DI: health assessment questionnaire disability index, WBC: white blood cell, Neutrophils: neutrophil count, Hb: Hemoglobin, Plt: Platelet, AST: aspartate aminotransferase, ALT: alanine aminotransferase, Cr: creatinine, eGFR: estimated glomerular filtration rate, MTX: methotrexate, TOF: Tofacitinib, BAR: Baricitinib, PEF: Peficitinib, UPA: Upadacitinib, FIL: Filgotinib, csDMARDs: conventional synthetic disease-modifying antirheumatic drugs, SASP: Salazosulfapyridine, IGU: Iguratimod, BUC: Bucillamine, TAC: Tacrolimus, *p <0.05,**p <0.01, and ***p <0.001.
